# Polyurethane Versus Calcium Alginate Dressings for Split-Thickness Skin Graft Donor Site: A Systematic Review and Meta-Analysis

**DOI:** 10.7759/cureus.20027

**Published:** 2021-11-30

**Authors:** Abdulmalik Alsaif, Mohammad Karam, Ahmed A Aldubaikhi, Abdullah Alghufaily, Khaled Alhuwaishel, Salah Aldekhayel

**Affiliations:** 1 Medicine, School of Medicine, University of Leeds, Leeds, GBR; 2 Medicine and Surgery, Walsall Healthcare NHS Trust, Birmingham, GBR; 3 Medicine and Surgery, Farwaniya Hospital, Ministry of Health, Kuwait City, KWT; 4 Medicine, King Abdullah International Medical Research Center, Riyadh, SAU; 5 Medicine, College of Medicine, King Saud Bin Abdulaziz University for Health Sciences, Riyadh, SAU; 6 Medicine, School of Medicine, University of Manchester, Manchester, GBR; 7 Plastic and Reconstructive Surgery, College of Medicine, King Saud Bin Abdulaziz University for Health Sciences, Riyadh, SAU

**Keywords:** split-thickness skin graft (stsg), calcium alginate, polyurethane, dressings, donor site

## Abstract

Herein, we compare the outcomes of polyurethane and calcium alginate dressings for split-thickness skin graft (STSG) donor sites.

A systematic review and meta-analysis were conducted with a search of electronic databases to identify all randomised controlled trials (RCTs) and observational studies comparing the outcomes of polyurethane dressing versus calcium alginate for STSG donor sites. Primary outcomes were pain intensity, convenience for staff and patients, and adverse effects (namely, excessive exudate, infection rate, and hematoma). Secondary outcome measures included the assessment of healing, dressing changes, cosmetic appearance, and cost. Fixed and random-effect models were used for the analysis.

Four RCTs enrolling 127 subjects were identified. There was no significant difference between polyurethane and calcium alginate in terms of pain intensity on Day 1 (mean difference (MD) 0.13, P = 0.80) and Day 5 (MD = 0.20, P = 0.38), as well as the ease of application (odds ratio (OR) = 3.08, P = 0.47). However, there was a statistically significant improvement in patient comfort, favouring the polyurethane group (OR = 44.11, P < 0.00001). In addition, no statistically significant differences were noted in terms of adverse effects between the two dressings. In terms of cost, the calcium gluconate dressing had an overall higher cost compared to polyurethane.

Polyurethane is a more favourable dressing compared to calcium alginate for STSG donor sites in terms of patient comfort, healing, and cosmetic outcomes. However, comparable results were noted in terms of pain intensity, ease of application, and adverse effects profile. Cost-effectiveness analysis studies are required to justify its routine use.

## Introduction and background

Several dressing options are used for donor sites of split-thickness skin grafts (STSG); yet, no gold standard exists. Dressings differ in their ability to optimize donor site morbidities, such as pain, infection, delayed wound healing, and exudate formation,^ ^in addition to their cost-effectiveness [1-5]. This study evaluates two common dressings used for STSG donor sites, namely, polyurethane and calcium alginate dressings. Calcium alginate dressings are known for their ease of application, ability to absorb exudates, and their haemostatic properties. However, the gel formed can become dry within a few days of the application, which can lead to pain and discomfort, potentially jeopardising the mobility and comfort of the patient [6]. On the other hand, polyurethane dressings are preferred for their ability to maintain a high degree of moisture which prevents their adherence to the wound bed, thus reducing pain, maintaining patient comfort, and supporting rapid healing without difficulty. Due to their flexibility, they also act as a second skin layer to contain wound exudate to prevent bacterial contamination and trauma to the wound [7-10].

Several randomised controlled trials (RCTs) have compared polyurethane and calcium alginate dressings on STSG donor sites, but there is currently no consensus in the literature [3, 11-13]. The current study reviews both dressings in a systematic review and aims to pool data from various RCTs in a meta-analysis.

## Review

Methods

A systematic review and meta-analysis were conducted as per the Preferred Reporting Items for Systematic Reviews and Meta-Analyses (PRISMA) guidelines [14].

Eligibility Criteria

All randomised control trials and observational studies comparing polyurethane dressing with calcium alginate dressing for STSG donor site were included. Polyurethane was the intervention group of interest and calcium alginate was the comparator. All patients were included irrespective of age, gender, or comorbidity status. Case reports, cohort studies, non-comparison studies, and non-English studies were excluded from the review process.

Outcomes

Primary outcomes included pain intensity, convenience for staff and patients, and adverse effects (excessive exudate, infection rate, and haematoma).

Secondary outcomes included assessment of healing, dressing changes, cosmetic appearance, and cost.

Literature Search Strategy

Two authors independently searched the electronic databases of Medical Literature Analysis and Retrieval System Online (MEDLINE), Excerpta Medica database (EMBASE), Emcare, Cumulative Index of Nursing and Allied Health Literature (CINAHL), and the Cochrane Central Register of Controlled Trials (CENTRAL). The last search was conducted on May 23, 2020. The search terms for our intervention of interest consisted of “split-thickness skin graft”, “STSG”, “donor site”, “polyurethane”, “calcium alginate”, “conventional”, and “routine.” All terms were combined with adjuncts of “and” as well as “or”. To extend the screening for eligible articles, the bibliographic lists were also reviewed of the relevant studies.

Selection of Studies 

The title and abstract of articles identified from the literature searches were assessed independently by two authors. The full texts of relevant reports were retrieved and those articles that met the eligibility criteria of our review were selected. Any discrepancies in study selection were resolved by discussion between the authors.

Data Extraction and Management 

A Microsoft Excel data extraction spreadsheet (Microsoft® Corp., Redmond, WA) was developed following Cochrane's data collection form for intervention reviews. Two authors independently extracted and recorded data.

Data Synthesis

The authors aimed to perform a meta-analysis for outcomes reported by at least two studies. Odds ratio (OR) was used for dichotomous variables whereas mean difference (MD) was used for continuous variables. Review Manager 5.3 and Microsoft Excel were used for data analysis. Meta-analysis was performed using fixed and random effects models. Reported outcomes were given in forest plots at 95% confidence intervals (CIs). Heterogeneity was assessed using the Cochran Q test (χ2) and it was used to quantify inconsistency by calculating I^2^, which was interpreted as follows: 0% to 25% (low heterogeneity); 25% to 75% (moderate heterogeneity); and 75% to 100% (high heterogeneity).

Methodological Quality and Risk of Bias Assessment

Two authors independently assessed the methodological quality as well as the risk of bias for articles matching the inclusion criteria. For randomised trials, Cochrane's tool for evaluating the risk of bias was used. Domains assessed included selection bias, performance bias, detection bias, attrition bias, reporting bias, and other sources. RCT studies are classified studies into low, unclear, and high risk of bias.

Results

Literature Search Results

**Figure 1:**
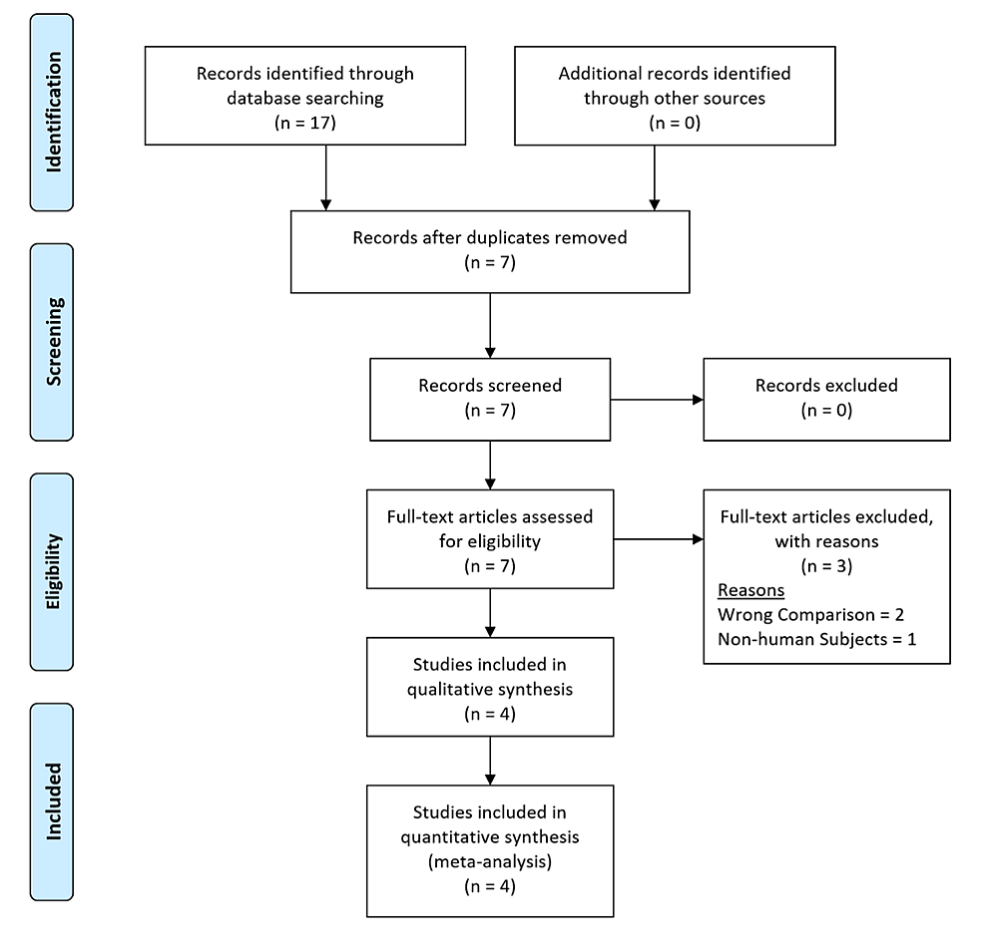
The PRISMA flow diagram details the search and selection processes applied during the overview In this article, PRISMA identified four studies to include in the meta-analysis [3, 11-13]. PRISMA: Preferred Reporting Items for Systematic Reviews and Meta-Analyses

In Figure 1, the literature search retrieved 17 articles in total which were reviewed by two independent authors to filter out duplicates, abstracts, review articles, studies without the intervention of interest, as well as those without comparative control groups and reports involving non-human subjects. Four RCTs were selected which met the eligibility criteria [3, 11-13].

Description of Studies

Baseline characteristics of the included studies are summarised in Table 1 [3, 11-13].

**Table 1:** Baseline Characteristics of the Included Studies [3, 11-13] NR: not reported; RCT: randomised controlled trials; UK: United Kingdom

Study (Year)	Journal, Country	Design	Mean Age (Range)	Sex (M:F)	Sample (Polyurethane: Calcium Alginate)	Interventions Compared
Vaingankar et al. [3]	Journal of Wound Care, UK	RCT	62.6 (11 - 90) years	3:13	16 (16:16)	Polyurethane dressing versus calcium alginate dressing
Terrill et al. [11]	Journal of Wound Care, Australia	RCT	71 (11 - 94) years	17:20	37 (19:18)	Polyurethane dressing versus calcium alginate dressing
Higgins et al. [12]	International Wound Journal, Australia	RCT	NR	16:20	36 (18:18)	Polyurethane dressing versus calcium alginate dressing
Läuchli et al. [13]	Dermatology, Switzerland	RCT	Polyurethane: 72.1 (35 - 95) years; calcium alginate: 78.6 (46 - 96) years	27:11	38 (19:19)	Polyurethane dressing versus calcium alginate dressing

Vaingankar et al. conducted a single centre prospective RCT that included 16 consecutive patients who required split-skin grafting harvest [3]. The study initially began with 20 patients but four were lost to follow-up and were not included in the final analysis. All patients had both types of treatment, so half of the donor site was covered with polyurethane dressings and the remaining half with calcium alginate.

Terrill et al. performed a single prospective RCT that enrolled 37 patients who required split-skin grafting to be harvested from their thigh to reconstruct a distant site defect [11]. The study initially started with 40 patients but three were lost to follow-up or failed to follow the protocol and were not included in the final analysis. A computer-generated randomisation chart assigned patients to either the calcium alginate dressing group (18 patients) or the polyurethane dressing group (19 patients).

Higgins et al. conducted a single centre RCT that included 36 patients who required a split-skin grafting procedure and had the graft harvested from the thigh [12]. Computer-generated randomisation sequence developed by an external agency assigned the patients to either the polyurethane dressing (18 patients) or the standard calcium alginate dressing (18 patients).

Läuchli et al. performed a single centre prospective RCT that included 38 patients who had an STSG donor site area of 12 - 300 cm^2^ [13]. Randomisation of patients took place by blinded allocation of treatment with 19 patients being treated with alginate dressings and 19 patients with polyurethane dressings.

Primary Outcomes

**Figure 2:**
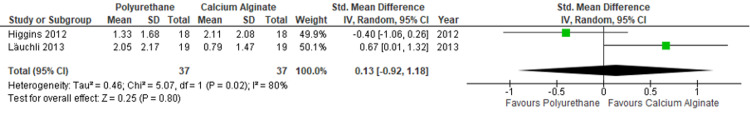
Forest plot for the mean difference of polyurethane dressing versus calcium alginate dressing – pain intensity at Day 1 Quantitative analysis showed no statistically significant difference in pain intensity at Day 1 in the polyurethane dressing compared with the calcium alginate dressing in two studies [12-13]. CI: confidence interval; df: degrees of freedom; IV: intravenous; SD: standard deviation; Std: standard

➣ Pain intensity: In Figure 2, pain intensity during the first day was reported in two studies enrolling a total of 74 patients [12-13]. There was no statistically significant difference seen in the standardised MD analyses which showed a lower intensity of pain for the calcium alginate group (MD = 0.13, CI = -0.92 to 1.18, P = 0.80). A high level of heterogeneity was found amongst the studies (I^2^ = 80%, P = 0.02).

In addition, Terrill et al. reported a significantly lower number of patients who experienced postoperative pain with the polyurethane dressing compared to the alginate group on both the first (21% versus 67%, P = 0.006) and second (17% versus 75%, P < 0.001) postoperative days [11]. Furthermore, the study also found a significantly lower score in the first two postoperative days for the polyurethane dressing compared to the alginate group (0 vs 2 on Day 1, 0 vs 2 on Day 2, respectively).

**Figure 3:**
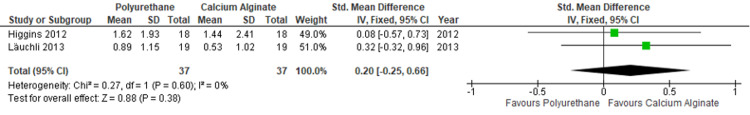
Forest plot for the mean difference of polyurethane dressing versus calcium alginate dressing – pain intensity at Day 5 The quantitative analysis showed no statistically significant difference in pain intensity at Day 5 in the polyurethane dressing compared with calcium alginate dressing in two studies [12-13]. CI: confidence interval; df: degrees of freedom; IV: intravenous; SD: standard deviation; Std: standard

In Figure 3, pain intensity on the fifth day was reported in two studies enrolling 74 patients [12-13]. There was no statistically significant difference seen in the standardised MD analyses which showed the lower intensity of pain on Day 5 for the calcium alginate group (MD = 0.20, CI = -0.25 to 0.66, P = 0.38). A low level of heterogeneity was found amongst the studies (I^2^ = 0%, P = 0.60).

Convenience for Staff

Convenience for staff was assessed through the ease of application and removal.

**Figure 4:**
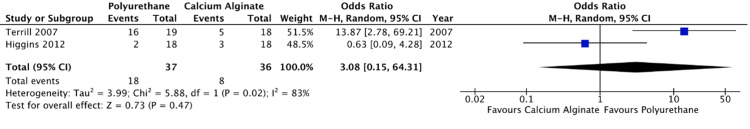
Forest plot for the odds ratio of polyurethane dressing versus calcium alginate dressing – ease of application by the staff The quantitative analysis showed no statistically significant difference in the ease of application in the polyurethane dressing compared with calcium alginate dressing in two studies [11-12]. CI: confidence interval; df: degrees of freedom; M-H: Mantel-Haenszel

➣​​​​​ ​​Ease of application by staff: In Figure 4, the ease of application by staff was reported in two studies enrolling 73 patients. There was no statistically significant difference seen in the odds ratio analyses which showed easier application by staff with polyurethane dressing (OR = 3.08, CI = 0.15 to 64.31, P = 0.47). A high level of heterogeneity was found amongst the studies (I^2^ = 83%, P =0.02) [11-12]. 

➣ Ease of removal by the staff: According to Terrill et al., 16 polyurethane dressings were rated by staff to have very easy removal compared to only two calcium alginate dressings [11]. Higgins et al. used the numeric rating scale (NRS) scale to assess the ease of removal, which showed no signiﬁcant difference (P = 0.79) in the staff perception of ease of dressing removal between polyurethane and calcium alginate dressings, with mean scores of 2.33 ± 1.41 and 2.22 ± 1.06, respectively [12].

Convenience for Patients

Convenience for patients was assessed through their comfort and satisfaction.

**Figure 5:**
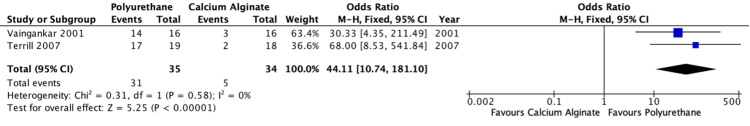
Forest plot for the odds ratio of polyurethane dressing versus calcium alginate dressing – patient comfort The quantitative analysis showed a statistically significant difference in patient comfort in the polyurethane dressing compared with calcium alginate dressing in two studies [3, 11]. CI: confidence interval; df: degrees of freedom; M-H: Mantel-Haenszel

➣ Patient comfort: In Figure 5, patient comfort was reported in two studies enrolling 69 patients. There was a statistically significant difference seen in the odds ratio analyses which showed a higher number of patients experiencing comfort with the polyurethane dressing (OR = 44.11, CI = 10.74 to 181.10, P < 0.00001). A low level of heterogeneity was found amongst the studies (I^2^ = 0%, P = 0.58). Additionally, Higgins et al. used the NRS to assess the comfort of patients and found no signiﬁcant difference (P = 0.79) between polyurethane (2.17 ± 1.25) and calcium alginate (2.06 ± 1.26) groups [12].

➣ Patient satisfaction: Terrill et al. and Higgins et al. assessed the satisfaction of patients [11-12]. Terrill et al. highlighted that 17 patients in the polyurethane group found the dressing to be convenient compared to only six patients in the control group [11]. In addition, Higgins et al. used the NRS to assess patient satisfaction, revealing that there was no signiﬁcant difference (P = 1.00) between polyurethane (2.39 ± 1.29) and calcium alginate (2.39 ± 0.61) groups [12].

Adverse Effects

**Figure 6:**
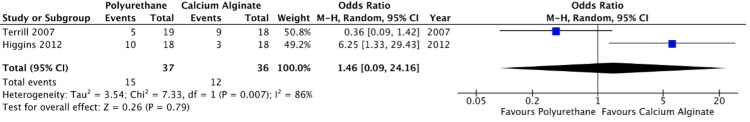
Forest plot for odds ratio of polyurethane dressing versus calcium alginate dressing – excessive exudate The quantitative analysis showed no statistically significant difference in the exudate reported in the polyurethane dressing compared with calcium alginate dressing in two studies [11-12]. CI: confidence interval; df: degrees of freedom; M-H: Mantel-Haenszel

➣ Excessive exudate: In Figure 6, excessive exudate was reported in two studies enrolling 73 patients [11-12]. There was no statistically significant difference seen in the odds ratio analyses which showed a lower rate of exudate for the calcium alginate dressing group (OR = 1.46, CI = 0.09 to 24.16, P = 0.79). A high level of heterogeneity was found amongst the studies (I^2^ = 86%, P = 0.007).

➣ Infection rate: Terrill et al. reported no cases of infections in both the polyurethane and calcium alginate groups [11]. In addition, Higgins et al. reported marginally fewer cases of infections in the former group (two cases) than the latter group (three cases) [12].

➣ Haematoma: Higgins et al. highlighted no incidence of haematoma in both groups [12].

➣ Associated symptoms: Terrill et al. demonstrated that the polyurethane group had fewer other symptoms than the control group, including pain (0 - 13%), hyperkeratinisation (31% - 46%), and itchiness (13% - 31%), without reaching statistical significance [11].

Secondary outcomes

Assessment of Healing

➣ Healing of donor sites: The percentage of donor sites healed was reported by Terrill et al. who found that 79% of the donor sites in the polyurethane dressing group had healed completely, compared to only 16% of the calcium alginate donor sites, with a statistically significant difference (P < 0.001) [11]. Terrill et al. also reported the median percentage of healed area that was 100% for the polyurethane dressing compared with 89.1% for the alginate dressing.

➣Time to heal: According to Vaingankar et al. and Higgins et al., there was no statistically significant difference between the two dressings in the meantime taken to heal [3, 12]. 

Time for Re-epithelialisation

Terrill et al. reported that the median time to complete re-epithelialisation was 14 days (range: 12 - 15) for the polyurethane dressing compared with 21 days (range: 8 - 23) for the alginate dressing, with a significant difference between the two groups (P < 0.001) [11]. However, Läuchli et al. found no significant difference in the time taken to full epithelialisation between the two dressings [13]. 

Dressing Changes

The mean time to the first dressing change was reported by Terrill et al. who found that the meantime for the polyurethane dressing group had a mean of 13 days (range: 8 - 16 days) versus 14 days (range: 10 - 20 days) for the calcium alginate group, with no significant difference between them (P = 0.34) [11]. Similarly, Higgins et al. also found that the polyurethane dressing required an earlier change, with a mean time of 5.50 days versus 8.11 days for the calcium alginate group, with a significant difference between the groups (P = 0.014) as summarised in Table 2 [12]. Terrill et al. reported that two cases in the polyurethane group and four cases with calcium alginate dressings required replacement of their dressings due to leakage [11]. In comparison, Higgins et al. showed 10 patients in the polyurethane group requiring more than one dressing change before Day 10 versus two patients in the alginate group [12].

**Table 2:** Mean Time Taken to First Dressing Change [11-12]

Study	Polyurethane dressing	Calcium alginate dressing
Terrill et al. [11]	13 days	14 days
Higgins et al. [12]	5.50 days	8.11 days

Cosmetic Appearance

According to Terrill et al., the skin appearance after the polyurethane dressing removal initially appeared slightly moist but dried within a couple of minutes to become a smooth, pale pink, epithelialized surface [11]. In contrast, the study reported that following the removal of alginate, the skin appeared dry, raised, and hyperkeratotic. Terrill et al. also assessed the scar using the Vancouver Scar Scale (VSS). At one month, the median score for vascularity for the polyurethane dressing was 2.5 (range: 1.5 - 3), compared with 3.5 (range: 3 - 4) for the alginate group, which was significantly greater (P = 0.014). In addition, the polyurethane dressing had lower median scar scores for height, pliability, and itchiness than the calcium alginate, but these were not statistically significant. At three months, a further assessment showed no significant differences in donor-site scarring between the two groups.

Cost of Dressing

The cost of applying each dressing type was reported by Vaingankar et al. and Terrill et al. as shown in Table 3 [3, 11]. The polyurethane dressing has a higher cost when equivalent sizes are compared. However, calcium alginate dressings require further reinforcements with gauze, wool, and bandage, meaning its overall cost is higher than the polyurethane dressing. 

**Table 3:** Cost Values of Polyurethane and Calcium Alginate Dressings [3, 11] AUD: Australian dollars; £: pounds sterling

Study	Cost of Polyurethane dressing	Cost of Calcium Alginate dressing
Vaingankar et al. [3]	A 7.5 x 7.5 cm dressing costs approximately £1.42	A 7.5cm x 12.5 cm dressing costs approximately £3.24
Terrill et al. [11]	A 20.0 × 20.3 cm dressing costs approximately AUD $16.00 (£7.11)	A 7.5 × 12 cm dressing costs approximately AUD $11.40 (£5.06)

Methodological quality and risk of bias assessment 

In Table 4 below, the quality of the RCTs included in the study was assessed in accordance with the Cochrane Collaboration’s Tool.

**Table 4:** The Cochrane Collaboration’s Tool was Used to Assess the Quality of the RCTs Included in the Study [3, 11, 12, 13] RCTs: randomised controlled trials; STSG: split-thickness skin graft

First Author	Bias	Authors’ Judgment	Support for Judgment
Vaingankar et al. [3]	Random sequence generation (selection bias)	Unclear risk	No information given
	Allocation concealment (selection bias)	Unclear risk	No information given OR justify your choice
	Blinding of participants and personnel (performance bias)	High-risk	Because both the intervention and control groups were located in a single patient, performing a blinded procedure was not possible in this study
	Blinding of outcome assessment (detection bias)	Low-risk	Although performing a blinded procedure was not possible in this study, both the intervention and control dressings were used in the same patient with similar donor sites, giving an optimal chance of a useful comparative assessment.
	Incomplete outcome data (attrition bias)	Low-risk	Consistency in numbers reported by the study and no missing data.
	Selective reporting (reporting bias)	Low-risk	All outcome data reported
	Other bias	Low-risk	Similar baseline characteristics in both groups
Terrill et al. (2007) [11]	Random sequence generation (selection bias)	Low-risk	Computer-generated randomization chart assigned patients to either the Kaltostat or Tegaderm Absorbent dressing group
	Allocation concealment (selection bias)	Unclear risk	No information given
	Blinding of participants and personnel (performance bias)	High-risk	Given the obvious difference in the dressings’ appearance, assessments were not blinded
	Blinding of outcome assessment (detection bias)	High-risk	Given the obvious difference in the dressings’ appearance, assessments were not blinded.
	Incomplete outcome data (attrition bias)	Low-risk	Consistency in numbers reported by the study and no missing data
	Selective reporting (reporting bias)	Low-risk	All outcome data reported
	Other bias	Low-risk	Similar baseline characteristics in both groups
Higgins et al. [12]	Random sequence generation (selection bias)	Low-risk	Patients were allocated to their group based on a computer-generated randomisation sequence
	Allocation concealment (selection bias)	Low-risk	The cards assigning patients to a treatment regimen were placed in a concealed envelope
	Blinding of participants and personnel (performance bias)	High-risk	Patients and staff were not blinded to the treatment allocation
	Blinding of outcome assessment (detection bias)	High-risk	Patients and staff were not blinded to the treatment allocation
	Incomplete outcome data (attrition bias)	Low-risk	No missing data
	Selective reporting (reporting bias)	Unclear risk	Insufficient information to permit judgment
	Other bias	Unclear risk	There isn’t enough information to assess whether an important risk of bias exists
Läuchli et al. [13]	Random sequence generation (selection bias)	High-risk	A randomised sequence generation hasn’t been used
	Allocation concealment (selection bias)	High-risk	Not described in sufficient detail
	Blinding of participants and personnel (performance bias)	Low-risk	blinding was likely effective
	Blinding of outcome assessment (detection bias)	Low-risk	blinding was likely effective
	Incomplete outcome data (attrition bias)	Low-risk	No missing data
	Selective reporting (reporting bias)	Unclear risk	Not described in sufficient detail
	Other bias	High-risk	There was a significant difference in the size of the STSG between the two groups

Discussion

The current systematic review and meta-analysis showed that polyurethane dressing of STSG donor sites has superior outcomes in patient comfort when compared to alginate dressing (Figure 5). This is supported by higher patient’s satisfaction with polyurethane dressing. However, no differences were detected in other outcome measures including pain, ease of application by staff, excessive exudate, formation of a hematoma, or infection (Figures 2-4, 6). Terrill et al. found that the polyurethane dressing group experienced less pain on postoperative Days 1 and 2 as compared to the alginate group [11]. Although the ease of application was similar between both dressings, more staff found polyurethane dressing to be easier to remove.

With regards to wound healing and cosmesis, studies were not eligible for meta-analyses. Descriptive data showed improved healing and higher scar assessment scores in polyurethane dressing as compared to alginate dressing; however, time to healing was similar in both. Polyurethane dressings seem to require earlier primary dressing with higher cost, however, cost-effectiveness studies need to be performed for better evaluation.

What is the ideal dressing for STSG donor sites remains debatable in the literature [15]. Alginate dressing is reported to have a high absorption capacity and high dehydration rate when compared to other types of wound dressings [16-17]. However, they can be painful to remove and cause higher hyperkeratosis of healed scars. Polyurethane dressings are shown to be more comfortable for patients and easier to be removed during dressing change [18]. Several reports showed that polyurethane dressing resulted in high healing rates and significantly reduced pain in treating skin graft donor sites [19-20].

The current study presents a systematic approach to produce a summary of the current evidence and assess its quality [3, 11-13]. Included studies were standardised based on the population and design. Intervention and comparison groups were homogenous across all the studies. All of these features aided in producing a non-biased comparative review. However, only four RCTs with a total of 127 subjects were included. This may not be sufficient to produce a definitive conclusion with a potential type 2 error to the study findings. Therefore, further high-quality RCTs are required to further evaluate the findings of the current study.

## Conclusions

Despite the limited number of studies, the results of this meta-analysis suggest that polyurethane dressings are more favourable compared to calcium alginate dressings in managing STSG donor sites as they are associated with improved patient comfort, healing, and higher scar assessment scores, with comparable pain intensity, ease of application, and adverse effects. Cost effectiveness analysis studies are required to justify their routine use.
